# Sensor-Assisted Assessment of the Tribological Behavioral Patterns of Al–SiCp Composites under Various Environmental Temperature Conditions

**DOI:** 10.3390/ma12234004

**Published:** 2019-12-02

**Authors:** Manivannan S, Senthil Kumaran S, Srinivasan Narayanan, Kathiravan Srinivasan, Alex Noel Joseph Raj

**Affiliations:** 1 Department of Mechanical Engineering, Karpagam Academy of Higher Education, Coimbatore - 641 021, Tamil Nadu, India; manivannan.s@kahedu.edu; 2 Department of Manufacturing Engineering, School of Mechanical Engineering, Vellore Institute of Technology (VIT), Vellore - 632 014, Tamil Nadu, India; senthilkumaran.s@vit.ac.in (S.K.S.); srinivasan.narayanan@vit.ac.in (S.N.); 3 School of Information Technology and Engineering, Vellore Institute of Technology (VIT), Vellore - 632 014, Tamil Nadu, India; kathiravan.srinivasan@vit.ac.in; 4 Key Laboratory of Digital Signal and Image Processing of Guangdong Province, Department of Electronic Engineering, College of Engineering, Shantou University, Shantou 515063, Guangdong, China

**Keywords:** aluminum, SiC_p_, stir casting, high-temperature wear, worn-out surface

## Abstract

Currently, the use of sensors and supporting technologies has become indispensable in the assessment of tribological behavioral patterns of composites. Furthermore, the current investigation focused on the assessment of the tribological behavior of the Al–SiCp composite for high-temperature applications. Moreover, the Al–SiCp composite was fabricated by adapting the liquid metallurgy route with varying weight percentages of SiC_p_ (x = 3, 6, and 9). Density, hardness, and high-temperature wear tests were performed to evaluate the hardness and tribological characteristics and properties of modern-day advanced composites. Moreover, the inclusion of SiC_p_ enhanced the advanced composite materials hardness from 60 HV to 110 HV due to a high degree of refinement of the α-phase. Subsequently, the fabricated samples’ wear behavior was assessed by varying the wear parameter viz. the applied load (20 N and 30 N) and sliding distance (250 m, 500 m, 750 m, and 1000 m) with the constant sliding velocity (0.45 m/s) for various temperatures (40 °C, 150 °C, and 250 °C). Moreover, the results revealed that the enhancement in the reinforcement percentage improves the wear resistance. Consequently, the wear rate decreased at 250 °C, possibly owing to the development of the oxide layers. Therefore, the occurrence of delamination and plastic deformation were evidenced in the wear-out surface, thereby depicting the prevalence of delamination and the abrasive wear-mechanism.

## 1. Introduction

Industry X.0 is pushing manufacturing and production businesses to move forward from conventional practices towards the use of progressive digital technologies to accomplish sustainable development. The emergence and advancement of day-to-day science and technology will augment the necessity for lightweight materials with unique characteristics compared to conventional materials. Aluminum-based alloys are used for fabricating automobile components like pistons, cylinder heads, cylinder blocks, among others, because of the alloys supreme specific strength and stiffness [1,2,3]. Furthermore, among the available Al alloys, Al–Si alloys exhibit various unique properties, including large strength-to-weight-rate, excellent castability, less thermal expansion, and high corrosion resistance. Moreover, such alloys exhibit weak wear-resistance that reduces their application in tribological environments (slippage under fretting, loading, degradation of material in sliding condition) [4,5,6,7]. This demand has prompted material researchers, product inventors, and manufacturers to explore the area of composite materials. Composite manufacturing was identified as the preeminent method to alter the required properties of the base material by adding suitable reinforcement particles. Amid the available composites, the metal matrix composite (MMC) has a comprehensive range of applications with the potential for use in the aerospace and automobile industries. The MMC is fabricated via various routes that include powder metallurgy, stir casting, squeeze casting, among others. In this work, the stir casting route was considered as the most economical method for the mass production of components [8,9,10,11,12]. These MMC’s are reinforced with several organic, carbides, and nitride-based materials that include graphite, graphene, silicon carbide, titanium nitride, among others, to enhance the performance of the base materials [13,14,15,16]. In general, these carbide-based ceramic particles are widely deployed as reinforcement agents to enhance the hardness and wear behavior of the Al MMC. In the existing literature, the carbide-based reinforcements are employed as agents to enhance the base material’s wear behavior. Moreover, the SiC_p_-based hybrid Al MMC was developed by Daniel [17] and his coworker to augment the high temperature wear performance of the Al alloy. Subsequently, the authors studied the wear parameters’ performance, including load velocity, sliding-distance, SiC_p_ particle size, and temperature of the developed MMC. They found that increases in temperature decreased the wear resistance of the MMC. However, decreases in particle size and applied load showed an incremental trend in wear resistance. It was also found that the weight percentage of SiC_p_ acted as a significant parameter in governing the wear behavior of MMC. 

Smrutiranjan et al. [18] devised a stir casting scheme for fabricating an Al–SiCp composite with a constant wt.% of SiC_p_ (7.5 wt.%). Moreover, they investigated the wear behavior of the composite with variations in environmental conditions that included dry, alkaline, and aqueous medium with varying wear parameters of applied load and speed. They observed that the wear rate increased with the increment in wear parameters under different environmental conditions and they concluded that the wear rate was maximum for the samples analyzed under an alkaline medium. Khan et al. [19] developed a SiCp reinforced Al MMC by adopting a stir casting scheme for assessing the wear characteristics of the developed MMC. Consequently, they observed that there was an increase in hardness and wear performance. However, the addition of SiC_p_ showcased a declining trend in tensile and fatigue strength. They also revealed that the wear rate was minimal for Al MMC under saline and acidic mediums. Rouhi et al. [20] performed a comparative study to assess the wear characteristics of the hybrid-based MMC (SiC_p_ and MoS_2_). Additionally, the experimental outcomes demonstrated that the inclusion of SiC_p_ up to 10 vol.% and MoS_2_ up to 2 vol.% exhibited better wear resistance, thereby leading to a lower wear-rate.

Based on the exhaustive literature survey, it is evident that the inclusion of SiC_p_ augments the base materials wear performance. However, there are only a few works on the wear behavior of composites under elevated temperatures. In this research, the Al MMC materials dry sliding wear characteristics were explored under distinct temperature ranges. There is a current need to explore the wear behavior of Al MMC under various environmental temperatures to develop a more comprehensive range of applications. Based on the facts above, the present work dealt with the fabrication of Al MMC under varying wt.% SiC_p_ (0, 3, 6, and 9) and carried out a detail investigation of the wear behavior of developed composites under different environmental conditions.

## 2. Materials and Methods

**Figure 1:**
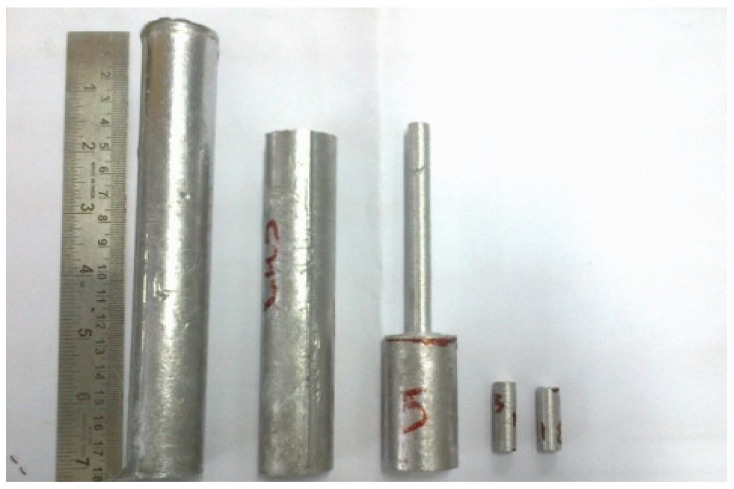
Illustrating the cast samples of the developed metal matrix composite (MMC).

Hyper eutectic Al–Si alloy with 13.2% Si was melted in a graphite crucible in an electric resistance furnace and held at 850 °C for an hour to attain a homogeneous composition. After degassing with 1.0 wt.% solid hexachloroethane to remove porosity, the melt was stirred for 5 min at frequent intervals for an hour after adding preheated SiC_p_ ceramic particles. Then the melt was poured into a cast-iron mold preheated at 300 °C. The cast samples were 160 mm long and 26 mm wide, and the percentage of SiC_p_ varied from 2.0 to 6.0 wt.% is shown in Figure 1. The Al–SiCp composite specimens were cut into small pieces (10 mm × 100 mm) for metallography studies. The Keller’s reagent (2.5% HNO_3_, 1.5% HCl, 1% HF and 95% H_2_O) etchant was used in this study. The experimental composite specimens used in the present work were analyzed for composition using an optical emission spectrometer (OES) (Model ARL 3460 from Thermo Electron Corporation, Switzerland). Table 1 also shows the compositions of the Al–SiCp composites that were studied in the current research. The microstructural analysis was characterized using the DIC Leica optical microscopy (Model No: DM750M) and scanning electron microscopy (Hitachi SEM, NIT Trichy, Tamil Nadu, India). The current study added different levels of SiC_p_ to the Al–Si alloy to understand the grain refining efficiency.

In the current study, different levels of SiC_p_ could successfully suppress the formation of coarse dendritic α-Al grains into fine equiaxed grains. The grain refining efficiency of different levels of SiC_p_ like x = 3.0, 6.0, and 9.0 wt.% was influenced by the formations of heterogeneous nucleation in the melt. The composite contained the fine SiC_p_ particles during grain refinement with the Al–Si alloy; only SiC_p_ particles acted as heterogeneous nucleating sites for the Al.

**Figure 2:**
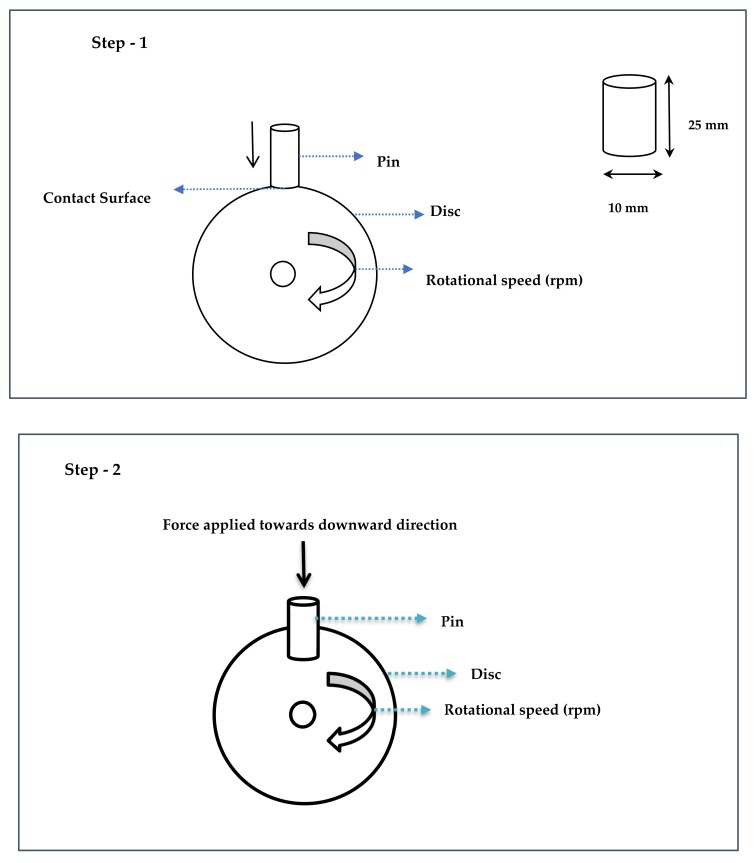

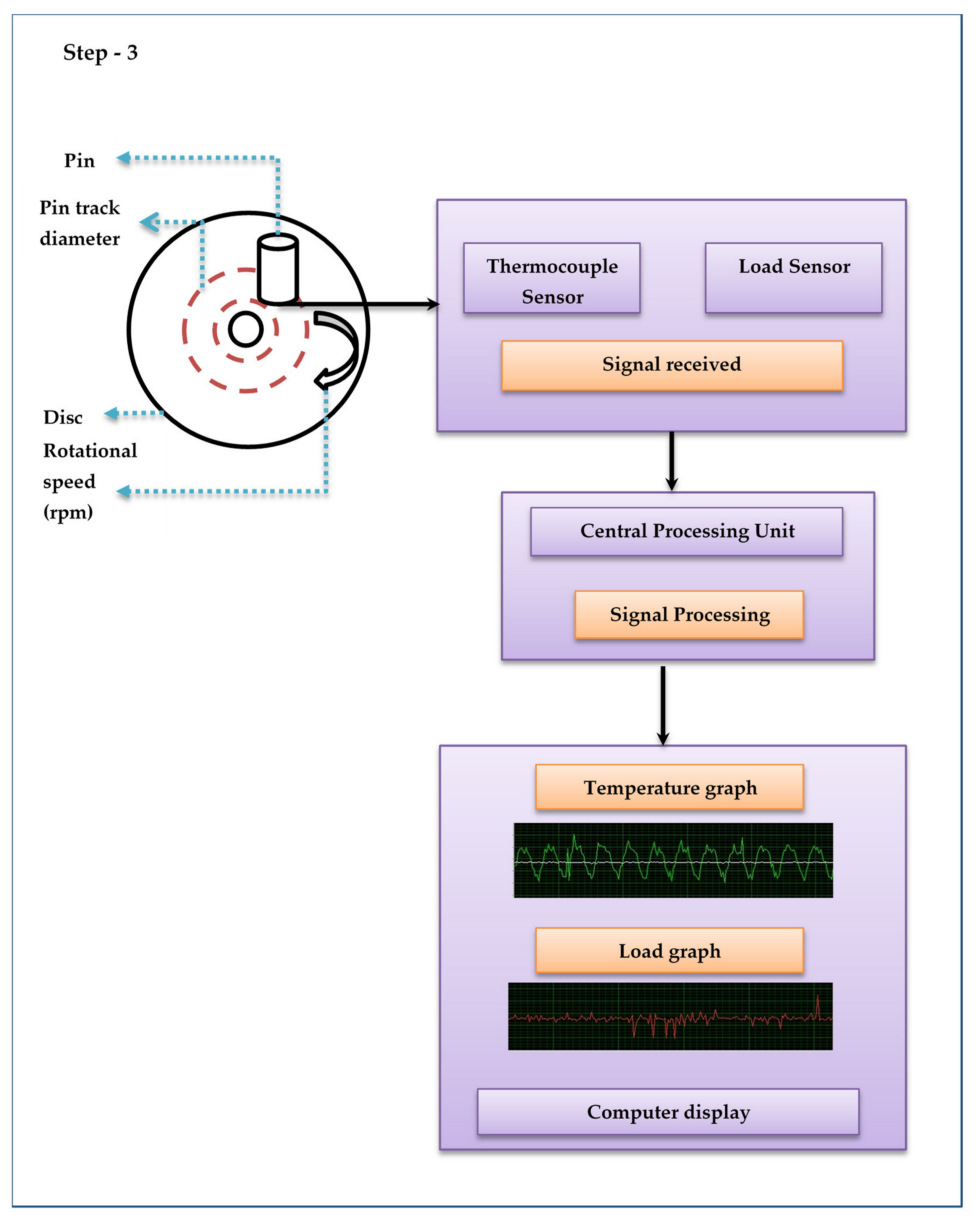
Step 1–3 for temperature and load prediction using different sensors in the tribology test.

Figure 2 shows the precise control of temperature and load prediction during the wear tests performed by utilizing the pin on a disc machine, where step 1 is influenced by the pin and disc arrangement position for real brake pad application in any automobile. While testing the MMC sample, the pin is stationary when the disc is rotated during contact. The pin is moved in a downward direction towards the direction of rotation of the disc position in the step 2 position. When the contact of the pin moves toward the disc, an enormous amount of heat is produced due to friction. The pin creates a track diameter on the disc upon contact of the two mating parts, and it drives higher heat dissipation during the respective period. The load is also changed when the pin acts on the disc while testing the sample. The corresponding sensor is mounted on the bottom of the pin and disc location, and simultaneously the heat and load are continuously monitored using a temperature and load sensor. The predicted value of the temperature and load is recorded and then processed by the CPU, followed by presentation using the LabView software.

## 3. Material Characterization

The microhardness tests of the fabricated samples were performed using Vickers microhardness testing machine. The applied load during the test was 0.3 kg, with a dwell time of 15 s. The Vickers hardness number (VHN) was calculated from Equation (1).

VHN = (1.854 P)/D^2^ (1)

where P = applied load, kgf, D = average length of diagonals, mm.

The density tests were conducted using a sartorius electronic balance. High-temperature wear tests were performed on the casted sample pin on the disc apparatus that consisted of the loading panel and controller. The rotating disc was fabricated with carbon steel of diameter 80 mm and hardness of 200 HV. The samples were held stationary, and the required normal load was applied using a lever mechanism. The test was conducted at three different temperatures 40 °C, 150 °C, and 250 °C under two different loading conditions, i.e., 20 N and 30 N, at a fixed sliding velocity of 0.45 m/s. Wear loss of the specimen was measured at four different sliding distances, i.e., 250 m, 500 m, 750 m, and 1000 m. The pin height was kept at 25 mm, and the diameter was fixed at 8 mm. Wear rate was assessed by measuring the mass loss in the specimen after each wear test and the mass loss, Δm, in the specimen was measured using an electronic weighing machine having a least count of 0.1 mg. Wear rate, which relates to the mass loss (Δm) and sliding distance (L), was calculated using Equation (2),

**W = Δm/L** (2)

## 4. Result and Discussion

**Figure 3:**
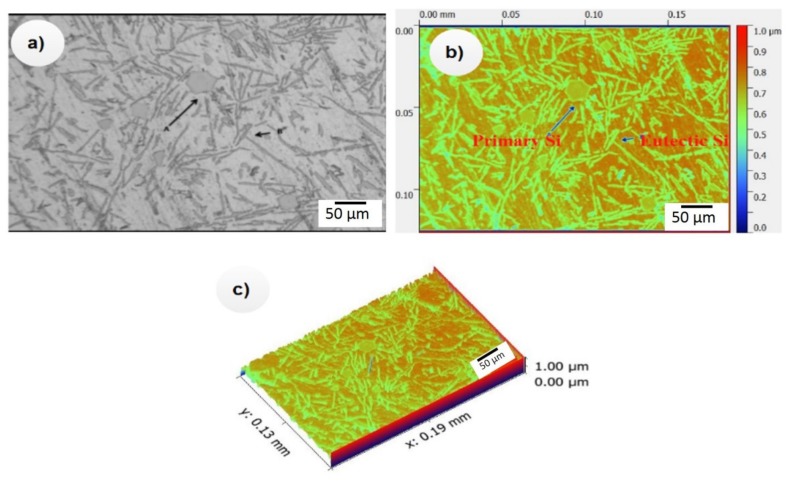
(**a**–**c**) Microstructure of the developed Al–Si alloys.

**Figure 4:**
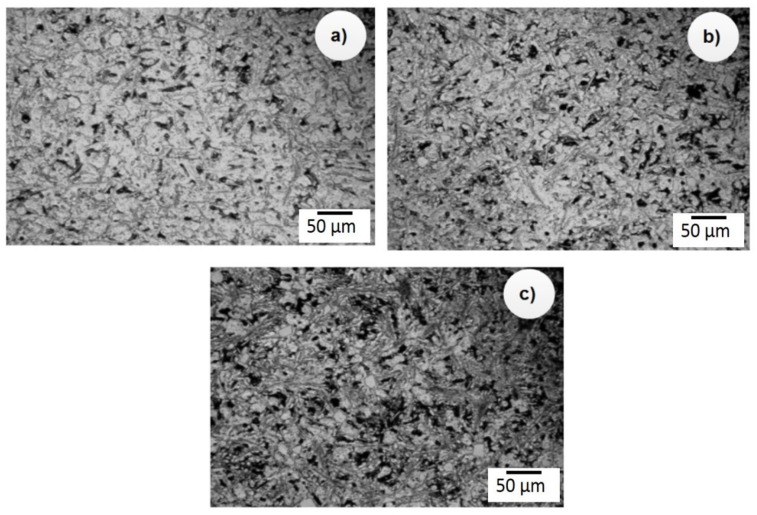
Microstructure of (**a**) Al-13%Si-2%SiC_p_ (**b**) Al-13%Si-4%SiC_p_ (**c**) Al-13%Si-6%SiC_p_.

To understand the mechanism of grain refinement of Al/SiC_p_ composites, the Al-13.2% Si alloy was modified with various levels of SiC_p_ like 2.0 wt.%, 4.0 wt.%, and 6.0 wt.% and the melt was poured into a water-cooled water mold of 32 K/s cooling at 5 min holding. The higher cooling rate was chosen to observe the SiC_p_ particles since a higher cooling rate allows the complete formation of SiC_p_ fine particles in the Al/SiC_p_ composites. The samples were subjected to DIC Leica optical microscopy (Figure 3) and SEM microanalysis (Figure 4) to observe the reaction of SiC_p_ with the nucleation particles, which improves wear resistance under various environmental conditions.

### 4.1. Microstructural Characterization

Figure 3a–c shows the microstructure of the developed Al–Si alloys. It could clearly show that the Al–Si alloy microstructure consisted of the eutectic mixture and primary Si. Figure 4a–c shows the microstructure of Al-13 wt.% of Si to which 2.0 wt.% SiC_p_, 4.0 wt.% SiC_p_ and 6.0 wt.% SiC_p_, respectively, was added. The microstructure analysis also confirmed that the addition of SiCp refined the eutectic mixture from a coarser to a fine fibrous structure. However, we observed several unidentified needles, formed either by the cleaning of the extracted particle or from Al_4_C_3_ possibly formed along with the SiC_p_ particles that reacted with the Al matrix. Generally, the Al_4_C_3_ was not easily identified, and Al_4_C_3_ is not easy to extract using any conventional procedure. In this study, we overcame the problem by developing a reclamation process that separated all the solid constituents such as reinforcement, reaction product, and insoluble metallic and non-metallic impurities in the composites melt. Using this technique, Al_4_C_3_ could be extracted even if it was formed in a small amount. However, the pits and porosities were not reflected in the density values because the density experiments were conducted in the unpolished samples. Moreover, the hardness results reflected only the surface and subsurface properties.

### 4.2. Density Analysis of the Developed Al MMC

**Figure 5:**
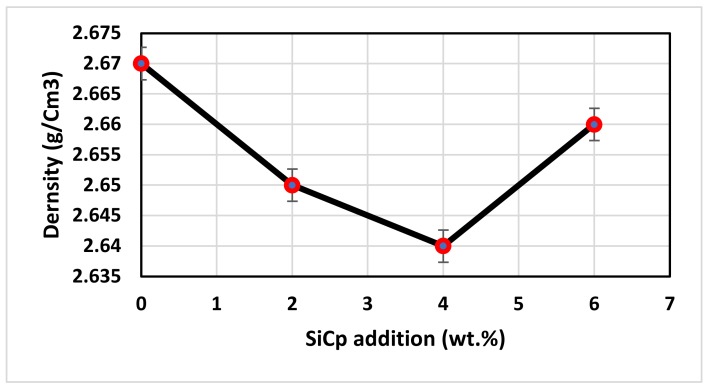
Influence of SiC_p_ on the Al–Si alloy density.

The experimental results from the density analysis are shown in Figure 5 showing a slight increment in aluminum density with every addition of SiC_p_ reinforcement. This increase in aluminum density was due to a higher density of the reinforced silicon carbide (3.21 g/cc). It could be noted from the plot that the density of unreinforced aluminum was near to 2.7 g/cc, which was similar to the actual density of the material that indicated poreless casting.

### 4.3. Hardness Analysis of the Developed Al MMC

**Figure 6:**
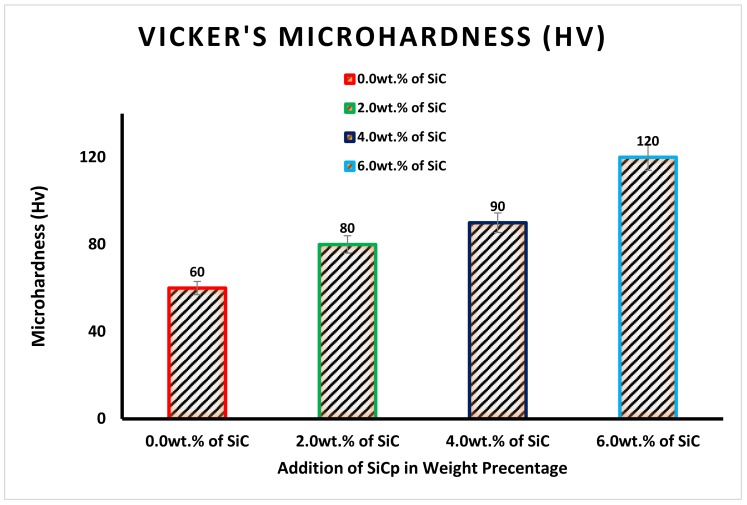
Influence of SiC_p_ on Al–Si alloy microhardness.

Microhardness results of the unreinforced base alloy and SiC_p_ reinforced composites are illustrated in Figure 6. The microhardness results indicated a positive trend from the addition of silicon carbide to the Al alloy resulting in increased hardness from 60 HV to 110 HV for 6% SiC_p_ addition, which was about 57%. This increment in hardness was due to hindrance offered by the reinforced SiC_p_ for dislocation motion during plastic deformation when the load was applied. This hindrance to dislocation motion under loading conditions results in an increased load requirement for plastic deformation that indicates the hardness increment [21,22].

### 4.4. Wear Behavior of a Developed, Fabricated Composite at Adverse Loading Conditions:

**Figure 7:**
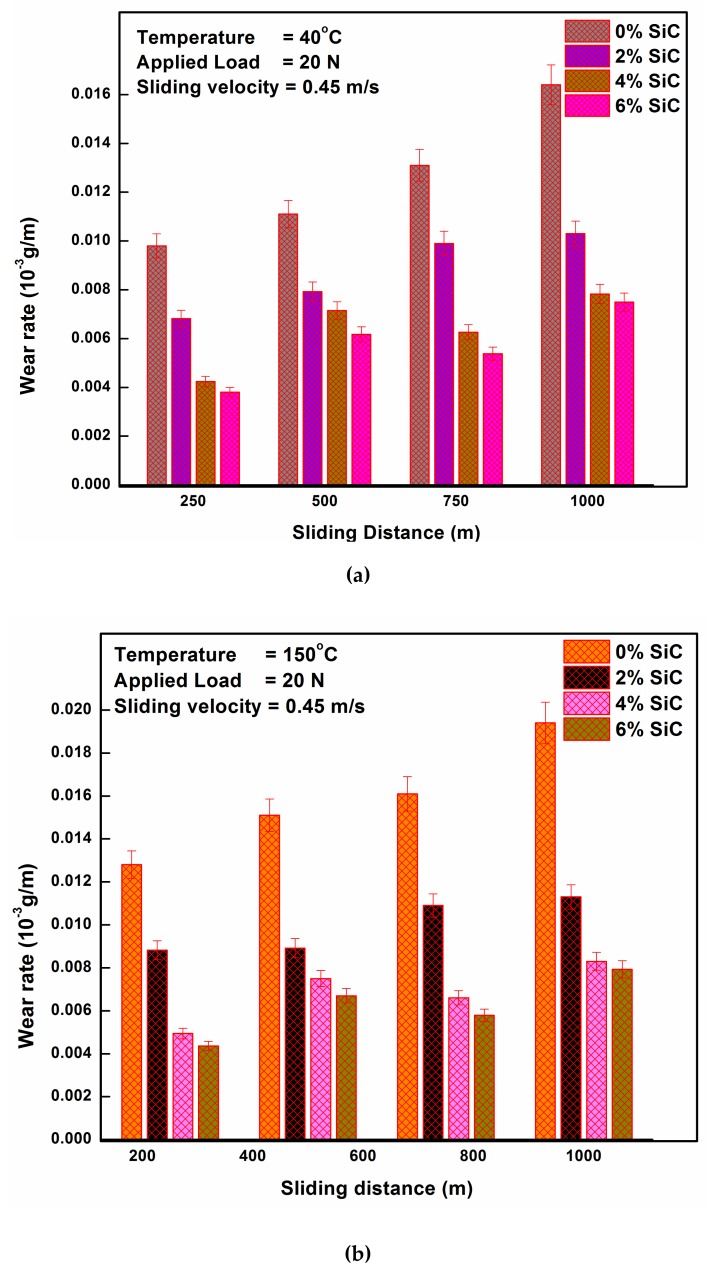

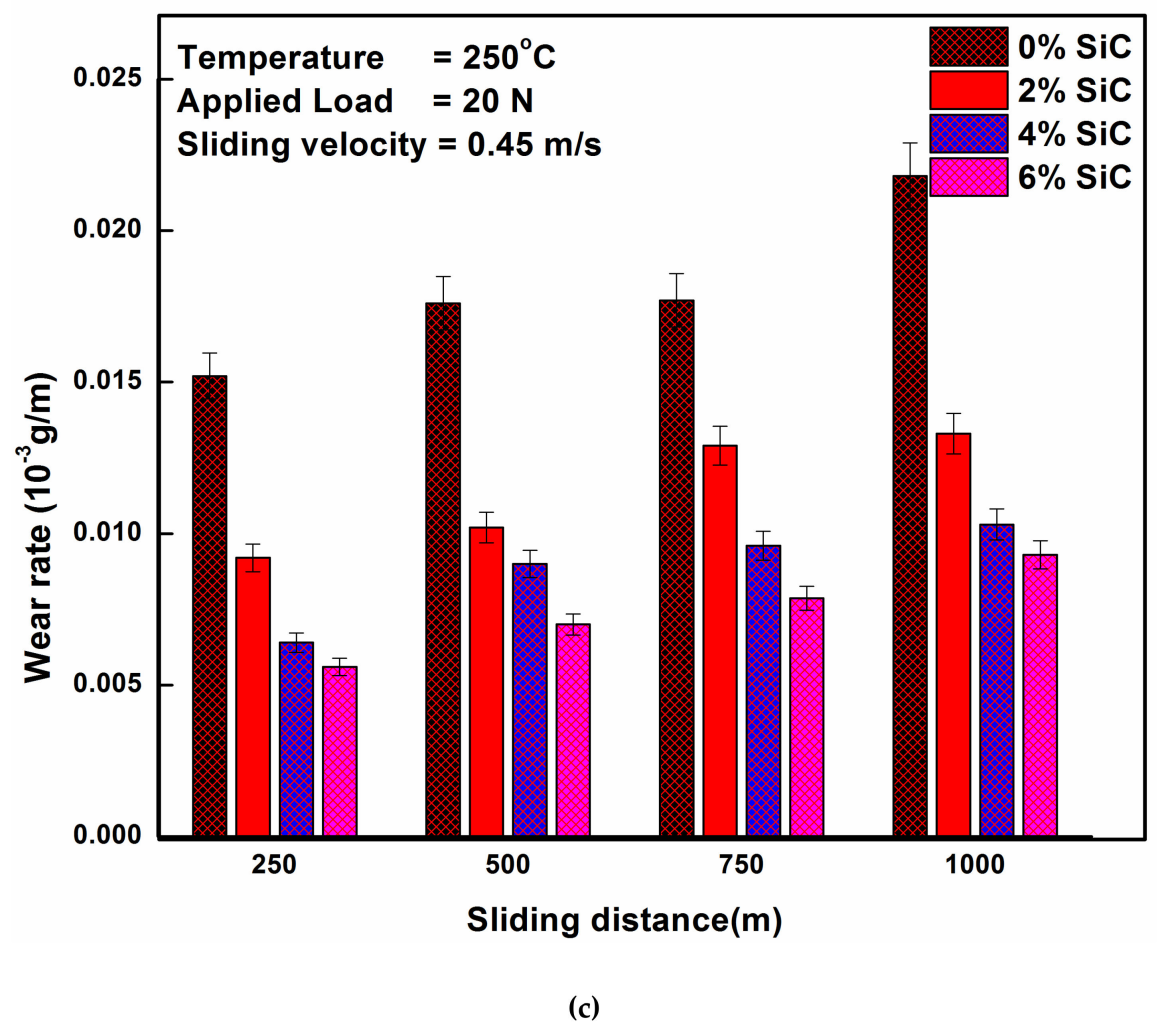
(**a**) Wear behavior of the Al MMC at 40 °C under a 20 N load, (**b**). Wear behavior of the Al MMC at 150 °C under a 20 N load, (**c**). Wear behavior of the Al MMC at 250 °C under a 20 N load.

Wear rate observed while testing the aluminum alloy with different SiC_p_ percentages for different sliding distances is given in Figure 7a. The temperature, load, and velocity were kept constant at 40 °C, 20 N, and 0.45 m/s, respectively. Figure 7b,c depict the wear rate for the same working conditions except for temperature, which was 150 °C and 250 °C, respectively. It is evident from Figure 7a–c that the inclusion of hard SiCp reinforcement into the soft aluminum matrix results in a decreased wear rate. That is, the wear-resistance capability of the aluminum increases with every addition of SiC_p_ irrespective of the temperature at which the test is conducted. Moreover, this may be because the hardness of the aluminum increases with SiC_p_ addition and it is a natural result that harder materials give higher resistance to wear. Since the load required for plastic deformation is higher for harder materials and hence the resistance to wear is also high. The wear rate increases with every increase in sliding distance, as a result of the increased temperature due to increased contact time with the counterpart [8,21,23].

This increased temperature softens the material resulting in more natural plastic deformation and, therefore, higher wear. Furthermore, the increase in the wear rate with sliding distance for unreinforced aluminum is higher than that of reinforced composites, which shows the capability of the developed MMC to withstand severe conditions [24].

While comparing Figure 7a–c, it is evident that the wear rate tends to increase with an increase in the temperature from room temperature (40 °C) to 250 °C. This outcome is because the increase in temperature softens the aluminum and reinforced composites, resulting in higher plastic deformation while sliding. Nevertheless, the increase in temperature causes a proportional increase in the wear rate [23,25].

**Figure 8:**
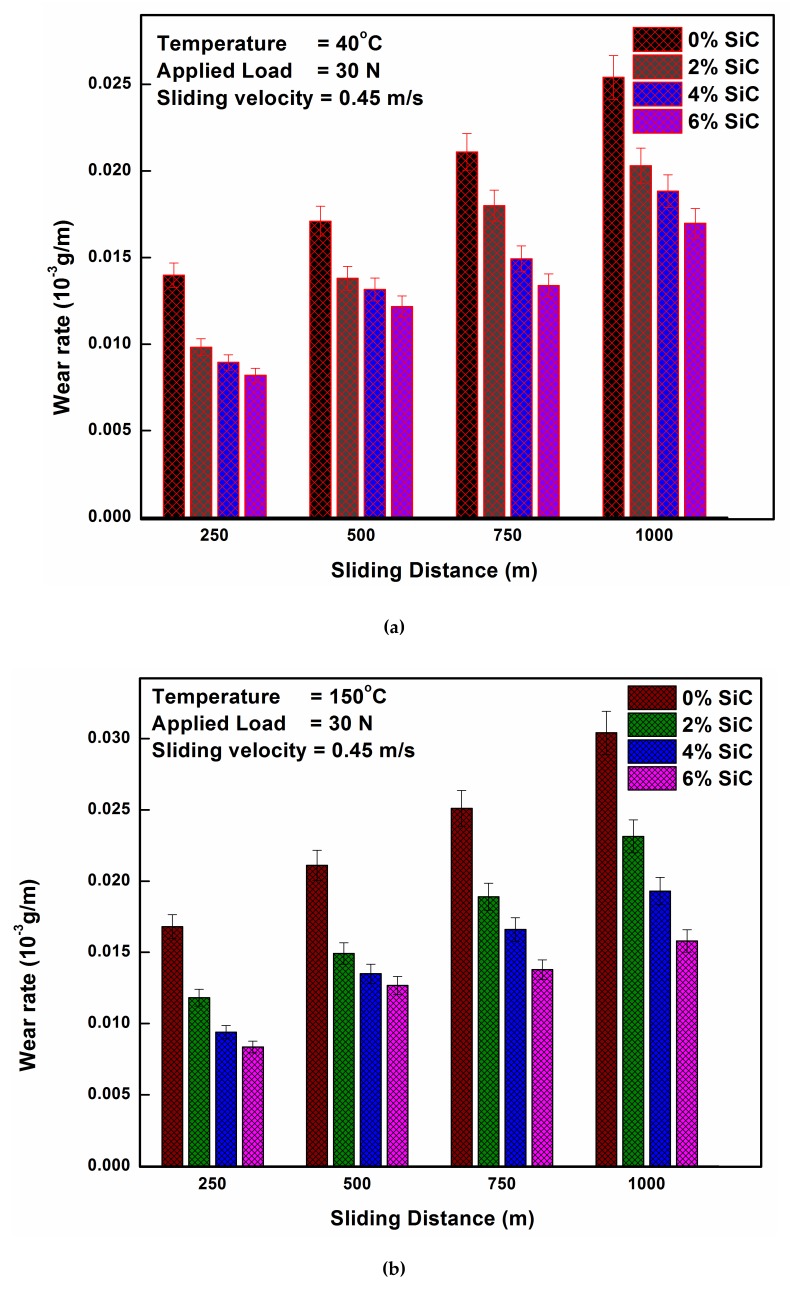

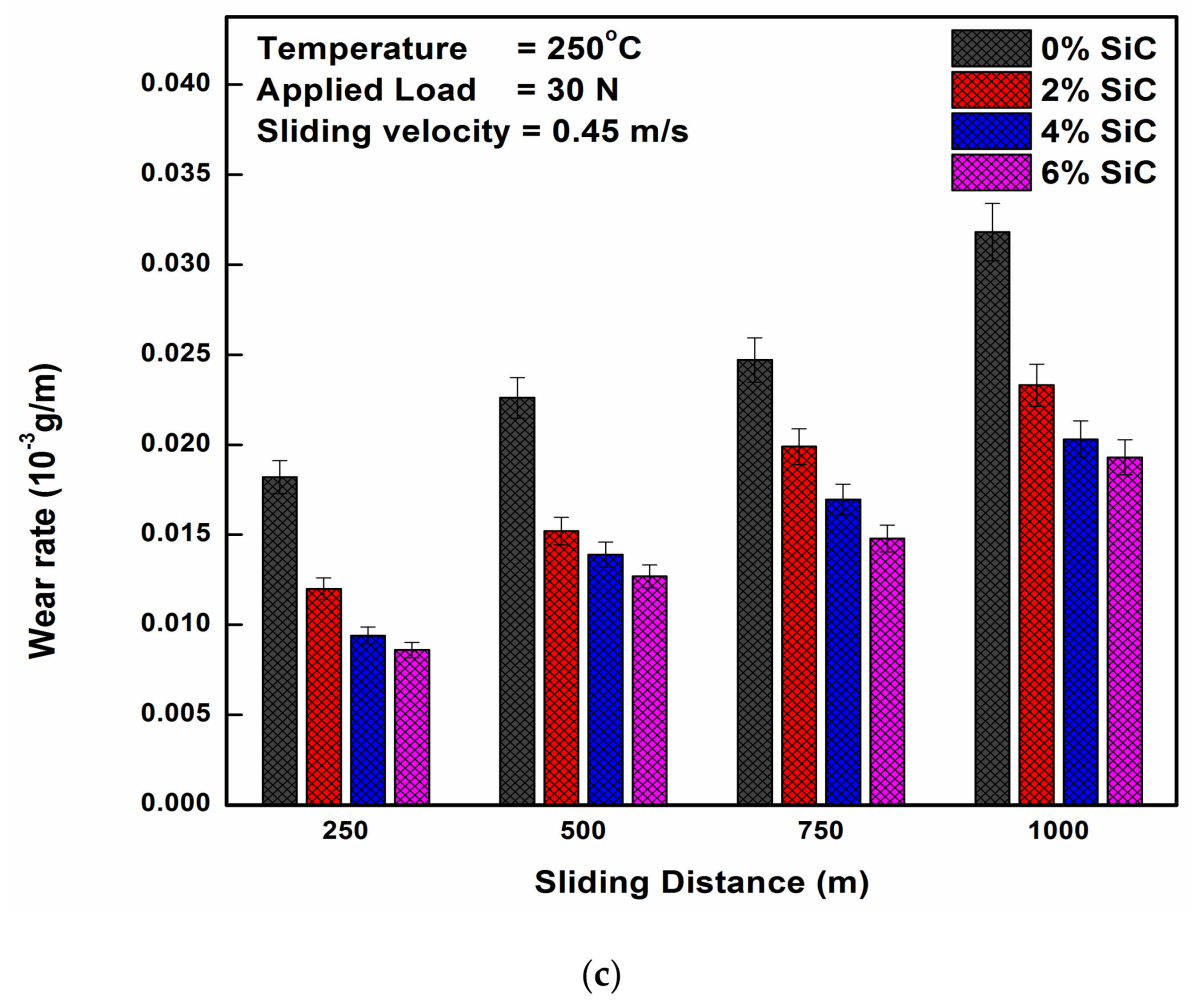
(**a**) Wear behavior of the Al MMC at 40 °C under a 30 N load, (**b**). Wear behavior of the Al MMC at 150 °C under a 30 N load, (**c**). Wear behavior of the Al MMC at 250 °C under a 30 N load.

Figure 8a–c shows the wear rate of the base and reinforced aluminum under different loading conditions while the load applied is 30 N. Comparing Figure 7 and Figure 8, we observed that the rate of wear while sliding increases with an increase in the load from 20 N to 30 N. This increased wear rate at higher loading conditions was due to the increased contact load and the friction between the two sliding parts that tends to increase heat generation at the interface. Moreover, this generated heat results in the softening of the material and higher wear [26,27,28,29,30,31,32].

### 4.5. Worn Out Surface Morphology

**Figure 9:**
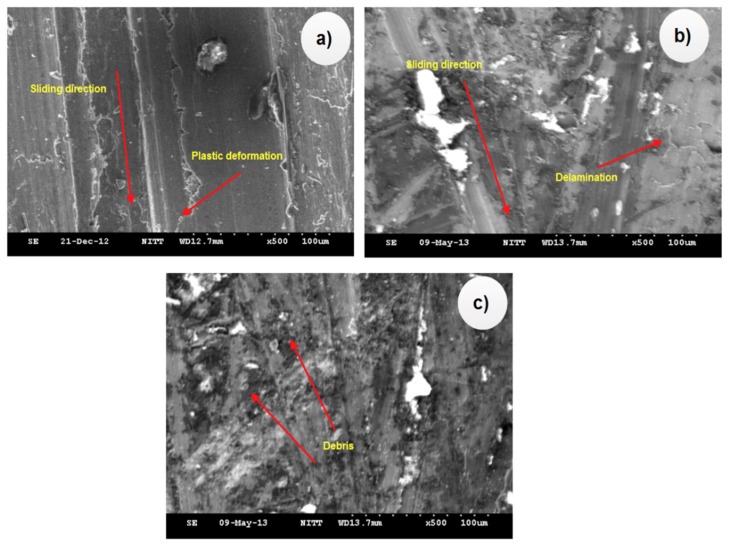
(**a**) The worn-out surface of the Al–Si matrix, (**b**) 6 wt.% SiC_p_ at an applied load of 20 N, (**c**) 6 wt.% SiC_p_ at an applied load of 30 N.

Figure 9a–c shows the worn-out surface of the tested Al–Si alloy under varied applied loads of 0 N, 20 N (6 wt.% SiC_p_), and 30 N (6 wt.% SiC_p_), respectively. The analysis confirmed the occurrence of abrasion and delamination wear over the worn-out surfaces on the pure Al–Si alloy. The Al-13Si alloy with 6 wt.% SiC_p_ under the 20 N and 30 N applied loads experienced the less abrasion, less debris, and delamination wear.

## 5. Conclusions

SiC_p_ reinforced Al MMC was successfully fabricated through a liquid metallurgy route. The influence of SiC_p_ over the functional properties of the composite was studied. We investigated the worn-out surface morphology of the samples under wear conditions. The following observations were made:

- The addition of SiC_p_ resulted in a drastic enhancement in hardness (~57%).
- Increments in SiC_p_ wt.% showed increments in wear resistance.
- The occurrence of abrasion and delamination wear was observed on the worn-out surface.
- The developed MMC can be practically used in high-temperature sliding applications where higher wear resistance is required. Advanced technologies for tribology behavioral pattern analysis shall be considered in future works [33,34,35,36].

The technical merit of this work is summarized as follows:

(a) The volumetric wear rate decreases with an increasing wt.% of SiCp, as well as a corresponding decrease in temperature.
(b) The wear resistance decreases at a higher temperature while it drastically increases with an increase in the wt.% of SiCp.
(c) The wear mechanism under the tested conditions was consistently adhesive in nature. The developed composite with a high wt.% of reinforcement agents exhibited abrasive wear mechanisms.

## Figures and Tables

**Table 1:** Chemical Analysis of Base Sample.

Elements	Si	Fe	Cu	Mn	Ni	Zn	Ti	Pb	Ca	Sn
Wt.%	13.2	0.72	1.06	0.219	0.0384	0.59	0.0419	0.079	0.0065	0.038
